# Bithionol is ineffective in a mouse model of *S. aureus* implant-associated osteomyelitis despite potent in vitro activity

**DOI:** 10.1038/s41598-025-08879-2

**Published:** 2025-07-06

**Authors:** Anders Marthinsen Seefeldt, Mikkel Illemann Johansen, Freja Winther Sillesen, Maiken Engelbrecht Petersen, Lars Østergaard, Rikke Louise Meyer, Nis Pedersen Jørgensen

**Affiliations:** 1 Department of Infectious Diseases, Aarhus University Hospital, Palle Juul-Jensens Blvd. 99, Aarhus N, Denmark; 2 Department of Clinical Medicine, Infectious Diseases, Aarhus University, Palle Juul-Jensens Blvd. 82, Aarhus N, Denmark; 3 Department of Surgery, Randers Regional Hospital, Skovlyvej 15, Randers, Denmark; 4 Interdisciplinary Nanoscience Center (iNANO), Aarhus University, Gustav Wieds Vej 14, Aarhus C, Denmark; 5 Department of Biology, Aarhus University, Ny Munkegade 114, Aarhus C, Denmark

**Keywords:** Experimental models of disease, Preclinical research, Translational research, Bacteriology, Biofilms, Clinical microbiology, Infectious diseases

## Abstract

Implant-associated osteomyelitis (IAOM) is a severe complication of the usage of orthopaedic implants. IAOM is difficult to treat and infection relapse is often due to insufficient treatment of bacterial persister cells. The anthelmintic drug bithionol is promising in combating persister cells - including *Staphylococcus aureus* persister cells. We investigated bithionol alone and in combination with antibiotics both in vitro and in vivo, using a murine IAOM model. *In vitro* experiments confirmed bithionol’s anti-persister activity against *S. aureus*, demonstrating significant CFU reductions in combination with daptomycin and moxifloxacin. In the murine IAOM model, moxifloxacin led to a small but significant reduction in bacterial load in bone of approx. log 0.5 CFU/ml. However, combining bithionol with antibiotics did not further reduce bacterial load in either bone or on implants. We subsequently demonstrated dramatically reduced activity of bithionol in the presence of albumin, suggesting low bioavailability *in vivo*. Despite promising in vitro results, bithionol’s efficacy against persister cells did not translate into improving treatment outcome the IAOM model. The study highlights the challenges in translating *in vitro* findings to *in vivo* outcomes, and future research into application of bithionol to treat bacterial infections must first address its bioavailability or investigate local delivery options.

**Supplementary Information:**

The online version contains supplementary material available at 10.1038/s41598-025-08879-2.

## Introduction

Infections of biomedical implants are characterised by formation of bacterial biofilms on the surface of the implant^1,2^. Biofilms are high density aggregates of bacteria embedded in an extracellular matrix that shields the bacteria from the immune system and provides a diffusion gradient, which can reduce the concentration of antibiotics to sublethal concentrations^3–5^. In the clinical setting, biofilm formation leads to infections that exhibit extreme recalcitrance towards antibiotic therapy^6,7^. This is most likely due to the contribution of several distinct biological mechanisms, that involves exchange of genetic material, prolonged antibiotic therapy and emergency of resistance^3,4,8^ .

Secondly, the biofilm milieu lead to the formation of persister cells in situ, a slow- or metabolically inactive subpopulation of bacteria that are transiently tolerant to antibiotics. Due to the inactive state of persister cells, conventional antibiotics are inefficient^9,10^. Once antibiotic therapy ceases, persister cells can switch back to an actively growing state and cause relapse of the clinical signs of infection^2,7,11–14^.

Implant-associated osteomyelitis (IAOM) is characterised by biofilm formation and is a feared complication^2,12,15^. Despite both surgery and prolonged antibiotics, treatment failure is frequent in part due to incomplete persister cell killing^2,8,10–13^.

One strategy to kill persisters is disrupting the bacterial cell membrane^9,10,16–18^. Recently, the anthelmintic drug bithionol demonstrated potent *S*. *aureus* anti-persister activity in vitro by disrupting the bacterial cell membrane and superiority in vivo in combination with gentamycin with approx. 1 log/CFU reduction in a murine thigh infection model^9^. As aminoglycosides are ill-suited for the treatment of staphylococcal IAOM, it is important to investigate bithionol in combination with other classes of antibiotics. The proposed mechanism of action of bithionol is inhibition of bacterial protein synthesis and translocation and by permeabilising the bacterial membrane^9,19^.

This study investigated the role of bithionol as a novel treatment strategy for *S. aureus* IAOM, with particular emphasis on killing persister cells. This was done by testing bithionol in combinations with two potent anti-staphylococcal antibiotics, daptomycin and moxifloxacin.

Daptomycin was selected based on a previous study demonstrating anti-persister activity against MRSA at concentrations exceeding x 10 MIC^20^ and moxifloxacin was chosen due to the central role of quinolones in treating implant-associated infections caused by staphylococci.

## Materials and methods

### Bacterial strains, culture and antibiotics

The clinical *S. aureus* isolate SAU060112 was used for both *in vitro* and *in vivo* experiments. SAU060112 was originally isolated from a prosthetic knee infection^21^. *S. aureus* cultures were cultivated on tryptic soy agar (TSA) and grown in tryptic soy broth media (TSB) (Sigma-Aldrich) overnight for 16–20 h (h) at 37˚C, 180 rpm. Additionally, a coagulase-deficient *S. aureus* double-knockout mutant (*S*. *aureus* ATCC 29213 *∆vWbp ∆coa*) was used for *in vitro* experiments that involved human plasma in TSB media^22^. Modified M9 buffer (mM9) (Sigma-Aldrich) was used for bacterial starvation^23^. mM9 contains M9 minimal salts (x1), 0.1 mM CaCl_2_, 2 mM MgSO_4_, 1 mM Thiamine-HCl, 0.05 mM nicotinamide, and 1 ml/L TMS3^24^. M9 solutions were adjusted to pH = 7.4 before autoclaving. Vitamins, salts and trace metals were sterile filtered and added to autoclaved M9 solution^23^. For all *in vitro* experiments involving daptomycin, 50 mg/L CaCl2 was added to the specific medium.

### Minimum inhibitory concentration (MIC) and minimal bactericidal concentrations (MBC)

MIC was determined by broth microdilution. Three overnight cultures of *S. aureus* were prepared and subsequently adjusted to OD_600_ = 0.01 (5 × 10^5^ CFU/ml). Compounds were dissolved in DMSO (max 1%) or NaCl (0.9%) and diluted in two-fold series in a 96-well microtiter plate. After 24 h of treatment at 37˚C, 50 rpm wells were visually inspected, and growth was measured by optical density (OD_600_). Growth inhibition was determined by no visible growth in wells and < 20% OD-value relative to OD-value of growth control. MBC was determined as the concentration with < 5 colonies after spotting out 10 µL from wells onto TSA and 24 h incubation at 37˚C.

### Checkerboard assay

Possible synergy or antagonism between compounds were tested using a checkerboard assay. Overnight cultures of *S. aureus* were adjusted to OD_600_ = 0.01 (5 × 10^5^ CFU/ml). The checkerboard assay was performed in a 96 microtiter well containing TSB and two-fold-dilutions of bithionol horizontally and either moxifloxacin or daptomycin in two-fold serial dilutions laterally. Bacteria were then added to all wells and allowed to grow for 24 h at 37˚C, 50 rpm before visual inspection and OD_600_ measurement. Experiments were performed in triplicates. Synergism, no interaction or antagonism was determined by calculating the Fractional Inhibitory Concentration Index (FICI). FICI 0.5-4.0 indicated additive/indifferent interaction, FICI < 0.5 indicated synergy whereas FICI > 4.0 indicated antagonism^25^.

### Minimal biofilm eradication concentration (MBEC)

Four *S. aureus* overnight cultures were prepared and adjusted to OD_600_ = 1 (5 × 10^7^ CFU/ml). PEG-lids (NUNC™ Immuno TSP lids, ThermoFischer Scientific (cat#445497)) were inoculated in adjusted culture for 60 min, 37˚C, 50 rpm. PEG-lids were then transferred to fresh TSB every 24 h and incubated at 37˚C, 50 rpm for a total of 48 h maturation before treatment start. Biofilm bacteria were treated 24 h with antibiotics dissolved in either DMSO or NaCl (0.9%) in a two-fold dilution series (concentration ranges of bithionol, daptomycin and moxifloxacin were 128 − 0.25 mg/L, 256 − 0.5 mg/L and 32-0.063 mg/L respectively and as for combination therapy experiments, bithionol concentration ranged from 32 to 0.063 mg/L whereas antibiotics daptomycin and moxifloxacin were held constant at 4 mg/L and 1 mg/L, respectively) at 37˚C, 50 rpm. PEG-lids were washed 2 × 1 min in fresh TSB media before sonication at 45 kHz for 10 min to release the biofilm. Biofilm bacteria were allowed to recover and grow for 24 h at 37˚C, 50 rpm before identifying the minimal biofilm eradication concentration (MBEC) as the lowest antibiotic concentration that did not lead to recovery of bacterial growth.

### Antimicrobial activity against persister cells

The persister cell assay has previously been described^23^. Briefly, three overnight cultures were prepared and subsequently diluted 1:1000 in fresh TSB and grown 16–20 h at 37˚C, 180 rpm. Cultures were washed by centrifugation at 13.150 x g for 10 min and resuspended in mM9 buffer, thus forcing the bacteria to starve to induce persister formation. Cultures were then adjusted to OD = 1 (5 × 10^7^ CFU/ml) in mM9 buffer. 20µL of washed culture were added to a microtiter plate, containing mM9 buffer and either bithionol at 0.01 ×, 0.05 ×, 0.1 ×, 0.25 ×, 0.5 ×, 1 ×, 2 × and 4 × MIC or in combination with antibiotics daptomycin or moxifloxacin at concentrations 0 ×, 5 × and 10 × MIC. The bacteria were treated 24 h at 37˚C. CFU was done from the three washed cultures before addition of antibiotics (T_0_) and after 24 h (T_24_). To wash off antibiotic remnants, 50 µL samples were centrifuged (as previously described) and resuspended in 50 µL mM9 buffer before 10-fold dilution series and plating on TSA were performed. CFU enumeration was done after 24 h incubation at 37˚C.

### Ethical statement

The animal studies were approved by the Animal Research Inspectorate under the Danish Ministry of Justice (Permission number: 2022-15-0201-01133) and followed the Danish act on animal experiments (LBK nr 474 of 15/05/2014 and BEK nr 2028 of 14/12/2020) as well as the EU directive 2010/63/EU on the protection of animals involved in scientific research. All animal experiments were performed in accordance with the relevant guidelines, including the ARRIVE 2.0 guidelines and the principles of the 3Rs. Animals were closely monitored with evaluation of activity levels and weighed every fourth day to prevent and minimize suffering (Supplementary Fig. 1). Animals were euthanized if humane endpoints were reached. Studies were conducted in close collaboration with veterinarians at the animal facilities at Skou building, Aarhus University. The study was not blinded.

### Antibiotics used *in vivo*

Bithionol was dissolved in Kolliphor HS-15 (Sigma-Aldrich)/ethanol 96%, 1:1 v/v and diluted 1:10 in saline to a concentration of 6 mg/ml = 30 mg/kg dose^9^. Daptomycin (“Cubicin”, Merck Sharp and Dohme) and Moxifloxacin (KRKA) were dissolved in NaCl (0.9%). Concentrations of daptomycin and moxifloxacin were 10 mg/ml and 1.6 mg/ml respectively which yielded doses of 50 mg/kg and 10 mg/kg respectively.

### Inoculation of tibial steel implants

Stainless steel insect pins (Benfidan, Nykøbing Mors, Denmark) were used as implants. An overnight culture was prepared and subsequently adjusted to OD_600_ = 0.1 (5 × 10^6^ CFU/ml) in TSB. Pins were placed in a 15 ml falcon tube, each containing 5 pins and 5 ml of adjusted *S. aureus* culture. Pins were followingly incubated stationary at 37^o^C for 24 h until use for surgery the next day.

### Implant associated osteomyelitis *in vivo* model in mice

60 6–8-week-old female C57bl/6j mice (Janvier Labs, Le Genest-Saint-Isle, France) were included in the study. At the time of inoculation, weight was between 17 and 21 g. Mice were acclimatised for a minimum of 7 days and subsequently housed at the animal facility at Department of Biomedicine, Aarhus University.

The surgical procedure was based on a model by Jørgensen et al.^5^. Briefly, mice were given analgesics prior to surgery (buprenorphine 0.05 mg/kg s.c.) and then anesthetized with isoflurane (induction 5%, maintenance 2%), oxygen (0.5 L/min) and air flow (1.2 L/min). The left hind leg was shaved, disinfected and the implant was pushed through the tibia at the proximal epiphysis. After insertion, the implant was bent in a U-shape and cut as close to the skin as possible. The skin and adjacent tissue was then managed to cover the implant ends. Buprenorphine (0.7 mg/kg) was administered in drinking water until 4 days post-surgery.

Following 7 days of infection, mice were randomised into six treatment groups with 10 mice in each group (pr. cage) by letter randomization. Groups were: (1) NaCl (0.9%) control (once daily); (2) bithionol (30 mg/kg/12 h); (3) bithionol (30 mg/kg/12 h) + daptomycin (50 mg/kg/24 h); (4) bithionol (30 mg/kg/12 h) + moxifloxacin (10 mg/kg/12 h); (5) daptomycin (50 mg/kg/24 h); (6) moxifloxacin (10 mg/kg/12 h). Treatment duration was 5 days and all drugs were administered intraperitoneally. No animals died or were excluded during the experiment. 36 h after last treatment dose, the mice were sedated and euthanized by cervical dislocation. Tibias including implants were carefully removed and kept on wet ice until sample preparation for bacterial quantification was performed immediately after.

### Post-mortem bacterial quantification

Implants were delicately separated from the dissected tibial bone and placed in Eppendorf tubes with 1 ml phosphate buffered saline (PBS). The tibial bone was cut in smaller fragments and transferred to Precellys MK28 hard tissue grinding tubes (Bertin Technologies, Saint Quentin, France) with 1 ml PBS and kept on wet ice. Bones were homogenized for 2 × 20 s at 5000 RPM in a bead beater (MagNA Lyser Instrument, Roche Diagnostics), transferred to wet ice to cool down for 2 min and homogenized again. Meanwhile, implants were sonicated in an ultrasonic bath (USCS1700 T, VWR, Westchester, PA, USA) for 5 min at 45 kHz and 110 W to release the biofilm on the implants. Implants were vortexed thoroughly in PBS before and after sonication. Now, 10-fold dilutions series of both sonicate and bone homogenate were made in PBS and 10 µl from each dilution row was plated out on TSA and incubated at 37^o^C for 24 h before enumerating CFU of all samples.

### Statistical analysis

Normality of data was assessed by QQ-plots. Parametric data were compared with either Student’s t-test or one-way ANOVA with Bonferroni post hoc correction and presented as means ± SD. Statistical analysis and graphs were made with GraphPad Prism (10.2.3, GraphPad software, San Diego, California, USA).

## Results

### Bithionol displays bactericidal activity against *S. aureusin vitro*

The isolate was susceptible to all tested antibiotics when comparing to the EUCAST breakpoints (Table 1). Bithionol inhibited growth at 2 mg/L (MIC) and eradicated bacteria (MBC) at 4 mg/L. Bithionol and daptomycin eradicated a 48-hour biofilm at a concentration of 8 mg/L and 4 mg/L respectively, whereas moxifloxacin achieved eradication at 0.25 mg/L (Table 1).

**Table 1:** MIC, MBC and MBEC determinations of different antibiotics and combinations.

Antibiotic	MIC (mg/L)	MBC (mg/L)	MBEC (mg/L)
Bithionol (BIT)	2	4	8
Moxifloxacin (MOX)	0.125	0.25	0.25
Daptomycin (DAP)	1	2	4
BIT + DAP (4 mg/L)	-	-	32
BIT + MOX (1 mg/L)	-	-	4

Brackets after antibiotics indicate the fixed concentration used for MBEC combination therapy experiments with bithionol. DAP = daptomycin, MOX = moxifloxacin. For MBEC experiments: *n* = 4.

### Bithionol combinations display additive antimicrobial activity and antagonistic effect against a biofilm

Prior to *in vivo* experiments, checkerboard assays and MBEC experiments were conducted to detect any potential synergism or antagonism between bithionol and daptomycin or moxifloxacin (Table 2). No synergy or antagonism was observed in any combinations using the checkerboard assay (Table 2).

Contrary to this, the MBEC experiments demonstrated an antagonistic effect of bithionol combined with daptomycin and moxifloxacin. Antagonism with daptomycin occurred at bithionol concentrations of 0.5–16 mg/L, while for moxifloxacin, this effect was observed at 0.5-2 mg/L (data not shown). Biofilm eradication was achieved at 4 mg/L of bithionol with moxifloxacin, and at 32 mg/L with daptomycin (Table 1).

**Table 2:** FICI-intervals from checkerboard assay determining interaction type between antibiotics.

Antibiotic	FICI-interval	Interaction
BIT + DAP (4 mg/L)	0.50–1.25	Additive
BIT + MOX (1 mg/L)	0.50-1.00	Additive

All combinations showed additive/indifferent interaction, meaning a fractional inhibitory index (FICI) interval from 0.5-4.0. FICI < 0.5 indicates synergy whereas FICI > 4.0 indicates antagonism. For checkerboard assays: *n* = 3.

### Bithionol displays biocidal activity against *S. aureus* persister cells

**Fig. 1:**
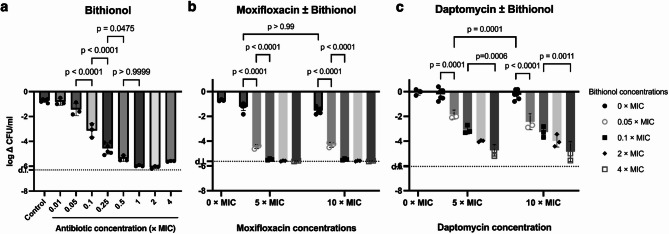
*S. aureus* persister cells treated with bithionol, DMSO or with combination of daptomycin or moxifloxacin for 24 h. a: Monotherapy with bithionol, ranging from 0.01 × MIC to 4 × MIC with DMSO (4%) as controls. b + c: Concentrations of bithionol ranged from 0.05 × MIC to 4 × MIC and concentrations of antibiotics were either 5 × MIC or 10 × MIC. ∆CFU/ml indicates log CFU/ml change. Dotted line represents detection limit (d.l.). Values represent mean ± SD. Brackets represent pairwise comparison (one-way ANOVA). *n* = 3–6.

Having established bithionol’s activity against metabolically active cells, we then evaluated the antimicrobial activity against persister cells. Based on previous data, bithionol was tested in a range of concentrations from 0.01 to 4 × MIC^9^. Overall, we observed a dose-dependent biocidal activity of bithionol against persister cells (Fig. 1a). Interestingly, under these conditions, biocidal activity was detected at 0.1 × MIC (leading to a reduction of log 3.13 CFU/ml (*p* < 0.0001, one-way ANOVA)), and the maximum biocidal effect leading to a log reduction of approximately log 6 CFU/ml was reached at 1 × MIC.

To test if the treatment efficacy against persister cells could be increased, bithionol was combined with either daptomycin or moxifloxacin at 5 × MIC and 10 × MIC. A range of concentrations of bithionol were chosen with the lowest dosages being ones, that would lead to a reduction less than log 3 CFU.

Monotherapy with daptomycin or moxifloxacin did not have any biocidal effect on persister cells at the tested concentrations (Fig. 1b + c). As expected, we also observed a dose-dependent biocidal effect of bithionol in this assay (Fig. 1b + c)). Combination with moxifloxacin augmented bithionol’s biocidal effect at a bithionol concentration of 0.05 × MIC, leading to log CFU reductions of 4.44 ± 0.16 when bithionol was combined with 5 × MIC moxifloxacin compared to 1.43 ± 0.51 for monotherapy (*p* = 0.0006, Student’s t-test). The same trend was observed when bithionol was combined with 10 × MIC moxifloxacin. At higher bithionol concentrations, the CFU reductions reached the detection limit (no colonies were observed) and we could therefore not compare the biocidal effect of combination therapy with monotherapy at these concentrations.

As for combinations with daptomycin, bithionol’s biocidal effect was only augmented at a bithionol concentration of 0.05 × MIC, leading to a log CFU reduction of 2.43 ± 0.64 when bithionol was combined with daptomycin at 10 × MIC compared to 1.43 ± 0.51 for monotherapy (*p* = 0.0202, one-way ANOVA). As for all other combinations, no augmented biocidal effect of bithionol was observed. Thus, bithionol alone seemed to drive the biocidal effect.

### Bithionol is ineffective against implant-associated osteomyelitis in mice

**Fig. 2:**
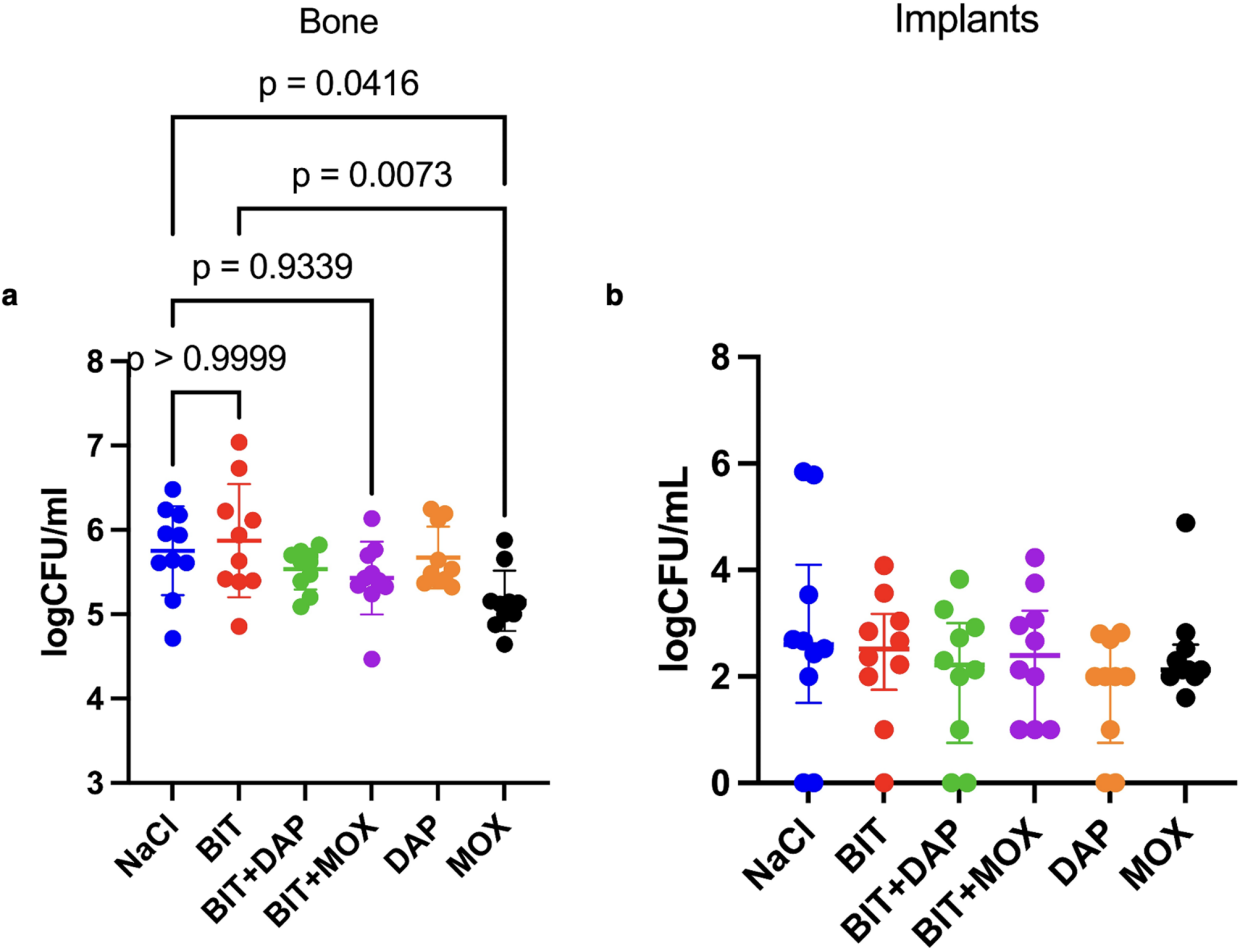
Bacterial load in bone fragments and from implants after 5 days of treatment in a murine model of *S. aureus* IAOM. Each datapoint represents the mean log CFU/ml of triplicates from each sample (bone or implant). a: bacterial load in bone fragments. b: biofilm bacterial load on implants. NaCl = NaCl (0.9%) in water, BIT = 30 mg/kg/12 h bithionol, MOX = 10 mg/kg/12 h moxifloxacin, DAP = 50 mg/kg/24 h daptomycin. Bars represent mean ± SD. *n* = 10/group. Brackets represent pairwise comparison (one-way ANOVA, *p* < 0.05 = significant).

Moxifloxacin was the only antibiotic to significantly reduce bacterial load in bone tissue compared to NaCl, albeit the effect was very small (log 5.16 CFU/ml vs. 5.75, *p* = 0.04, one-way ANOVA) (Fig. 2a). Bithionol + moxifloxacin did not reduce the bacterial load in bone tissue compared to NaCl (*p* = 0.93) or moxifloxacin (*p* > 0.99). Bithionol + daptomycin was comparable to bithionol + moxifloxacin (*p* > 0.99) as well as monotherapy with daptomycin (*p* > 0.99) and did not significantly reduce bacterial load in bone compared to NaCl (*p* > 0.99). Bithionol monotherapy was comparable to NaCl (*p* > 0.99).

Neither monotherapy nor combination therapy was efficient in reducing bacterial biofilm on the implants (Fig. 2b). Regardless of treatment with antibiotic alone or in combination with bithionol, the bacterial load on the implants of all treatment groups was not significantly different from that of the NaCl group.

### *In vitro* plasma binding reduces bithionol efficacy

The discrepancy in in vitro and in vivo findings led us to hypothesize that the fraction of free bithionol was reduced under in vivo conditions. To investigate this, we assessed the susceptibility of bithionol in the presence of CaCl_2_ (50 mg/L) due to its presence in both bone and plasma and given that daptomycin’s efficiency is contingent upon its presence. Since we added CaCl_2_ to the media when including daptomycin in in vitro combination therapy experiments with bithionol and because CaCl_2_ is abundant under in vivo conditions in the mice, we needed to assess whether bithionol’s biocidal action was altered by the presence of CaCl_2_. Furthermore, susceptibility was evaluated in the presence of human plasma, which required the use of a coagulase-deficient *S. aureus* strain.

The minimal concentration of bithionol that was able to kill persister cells after 24 h of treatment was 0.25 mg/L (= 0.125 × MIC). To test potential interaction between calcium and bithionol, CaCl_2_ was added to the solution. The minimum biocidal concentration for kill persister cells now increased from 0.25 to 1 mg/L, which suggests that CaCl_2_ inactivates bithionol or affects bithionol’s interaction with the bacterial cell membrane (Table 3).

**Table 3:** Minimum concentration of bithionol required to kill persister cells.

Antibiotic	Minimum biocidal concentration (mg/L)	
	mM9	mM9 + CaCl_2_
Bithionol	0.25	1

Performed as an MBC assay in mM9 buffer ± CaCl_2_ (50 mg/L) to promote persister cell phenotype. Introducing CaCl_2_ to the solution led to an increase in persister killing concentration from 0.25 mg/L to 1 mg/L.

**Table 4:** MIC and MBC values of bithionol against *S. aureus* 29213 *∆vwbp ∆coa*.

Media	MIC (mg/L)	MBC (mg/L)
TSB	2	8
TSB + 50% human plasma	> 32	> 32

Next, to investigate the impact of protein binding, MIC and MBC of bithionol against a coagulase-deficient *S*. *aureus strain (ATCC* 29213 *∆vWbp ∆coa)* was measured in TSB media containing human plasma. The addition of plasma led to an increase of both MIC and MBC to levels that were above the highest concentration used in the assay (Table 4). This indicates that the bioavailability of bithionol *in vivo* is low, possibly because it is bound to plasma proteins.

## Discussion

In this study we investigated the activity of bithionol against *S. aureus* under a range of in vitro conditions and in an established model of in vivo implant-associated osteomyelitis^5^. While bithionol was effective against *S. aureus in vitro*, particularly against persister cells, it failed to significantly reduce bacterial load on implants and bone in the murine model. This is in contrast with previous findings, in which bithionol in combination with gentamycin was able to reduce bacterial load in a deep-seated MRSA thigh infection mouse model^9^. While Kim et al. observed a reduction in thigh bacterial load from ~ 9 to < 8 log CFU/ml, no improvement in treatment outcome was observed for any combination with bithionol in our study. Only moxifloxacin treatment was efficacious, but this was only observed in bone and the reduction in bacterial load was low.

Follow-up in vitro experiments point to low bioavailability or activity of bithionol in plasma. Yet this cannot entirely explain our findings, as protein binding must also have occurred in the other *in vivo* studies using bithionol^9,18^. Given the low concentration of bithionol required to kill *S. aureus in vitro*, a further reduction of free bithionol at the site of infection might have occurred. As we investigated bithionol against implant-associated osteomyelitis, reduced diffusion into infected bone could explain the discrepancy between in vitro and in vivo efficacy. Poor diffusion of antibiotics into bone is a well-described issue and could potentially explain why a treatment with proven activity in either an abscess model or a peritonitis model, fails in an osteomyelitis model^12,26^. Under *in vitro* conditions, we observed decreased activity of bithionol in CaCl_2_ enriched media and as bone is calcium rich, this could further affect in vivo activity.

In our in vitro experiments, we observed notably greater activity of bithionol in vitro than Kim et al., as we observed > 3 log CFU reduction for persister cells at concentrations that were only a fraction of the inhibitory concentration for actively growing cells in laboratory media. Our data indicated that the fixed dosages of antibiotics used were above the MBEC value and we could therefore not conclude whether adding antibiotics enhanced the anti-biofilm activity of bithionol. These dosages should rather have been at sub-MBEC levels (4 and 0.25 mg/L respectively). As such, antagonism might play a part also in vivo since no effect of antibiotic combination was observed in vivo.

Our study has several limitations. Firstly, the small sample size in our *in vivo* studies is a limiting factor and was based on an assumption of greater efficacy than what was ultimately found. Increasing the treatment group sizes could allow detecting smaller but significant differences at the cost of using a very high number of animals to detect a difference with limited clinical impact. Secondly, treatment duration was very short compared to the clinical setting, in which treatment typically last for between 6 and 12 weeks. Finally, perhaps the dose was too low to treat an infection in bone as reduced penetration of conventional antibiotics into bone is often reduced^27^. Using local drug delivery mechanisms such as antibody-drug conjugates, nanoparticles or drug-eluding depot technology could be an alternative to simply increasing the dosage.

Finally, dose optimization could potentially overcome the lack of efficacy. To the best of our knowledge, there are no published data on bithionols pharmacokinetics in mice. Dosing in this study was therefore done in accordance with published data^9,18^. Given the good in vitro efficacy of bithionol more indepth pharmacological studies seem warrented.

Collectively, our in vitro findings revealed higher efficacy of bithionol compared to previous studies, leading us to anticipate a greater treatment efficacy in vivo. We hypothesize that a combination of inactivation by protein binding and an insufficient concentration of active bithionol at the site of infection accounts for the lack of effect.

## Conclusion

Bithionol showed great potential in vitro in eradicating persister cells, but both mono- and combination therapy failed to reduce bacterial load *in vivo*. Further studies are needed to establish if there is any true potential *in vivo*. Infections in bone do not appear as likely indications for a repurposing of bithionol.

## Electronic supplementary material

Below is the link to the electronic supplementary material.

Supplementary Material 1

## Data Availability

The datasets used during the current study are available from the corresponding author on reasonable request.
