# Supplementary material for: Bithionol is ineffective in a mouse model of S. aureus implant-associated osteomyelitis despite potent in vitro activity
Source: Sci Rep. 2025 Jul 6;15:24156. doi: 10.1038/s41598-025-08879-2 (PMC12230103; doi:10.1038/s41598-025-08879-2)
Supplement: Supplementary file 1 — Supplementary Material 1 [file 41598_2025_8879_MOESM1_ESM.pdf]

Supplementary

Bithionol and antibiotics were well tolerated in animals

Animals were monitored daily with evaluation of activity levels and weighed every fourth day to ensure that no animals violated pre-defined humane endpoints. No animals suffered from significant weight loss (Supplementary figure 1) or reduced activity levels during the study.

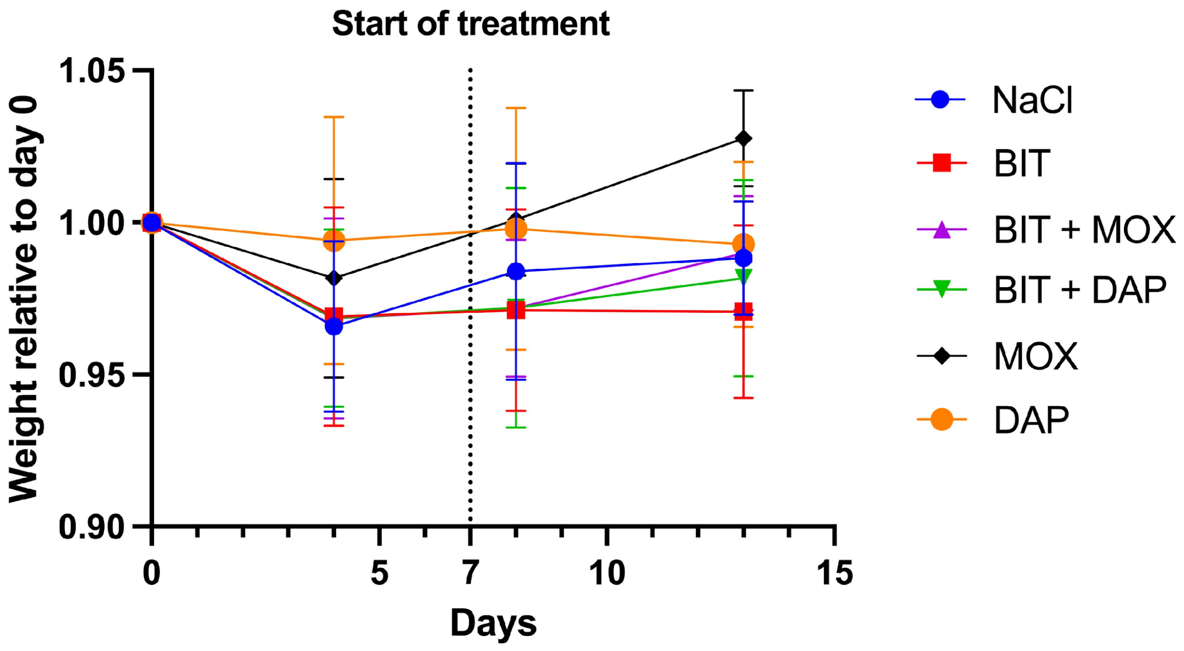

Supplementary figure 1. Relative weight changes of mice during study period of 14 days. Symbols represent mean with SD for each treatment group normalised to baseline value (day 0). BIT = bithionol, MOX = moxifloxacin, DAP = daptomycin.
